# Hybrid floral scent novelty drives pollinator shift in sexually deceptive orchids

**DOI:** 10.1186/1471-2148-10-103

**Published:** 2010-04-21

**Authors:** Nicolas J Vereecken, Salvatore Cozzolino, Florian P Schiestl

**Affiliations:** 1 Evolutionary Biology & Ecology, Free University of Brussels/Université Libre de Bruxelles, Avenue FD Roosevelt 50 CP 160/12, B-1050 Brussels, Belgium; 2 Institute of Systematic Botany, University of Zürich, Zollikerstrasse 107, CH-8008 Zürich, Switzerland; 3 Department of Structural and Functional Biology University of Naples "Federico II" Complesso Universitario Monte Sant'Angelo Via Cinthia, Build 7, I-80126 Napoli, Italy

## Abstract

**Background:**

Sexually deceptive orchids of the genus *Ophrys *attract their pollinators, male insects, on a highly specific basis through the emission of odour blends that mimic the female sex pheromone of the targeted species. In this study, we have investigated a contact site between *Ophrys arachnitiformis *and *O. lupercalis*, two sympatric orchid species that are usually reproductively isolated *via *the exploitation of different pollinator "niches", but occasionally hybridise despite their apparent combination of ethological and mechanical isolation barriers. In particular, we have investigated the extent to which these *Ophrys *hybrids generate "emergent" combinations (i.e. novel and unpredictable from the parents' phenotypes) of floral traits, and how these phenotypic novelties, particularly the odour blends emitted by the flower, could facilitate the invasion of a novel pollinator "niche" and induce the rapid formation of reproductive isolation, a prerequisite for adaptive evolutionary divergence.

**Results:**

Our chemical analyses of floral scents show that the *Ophrys *F1 hybrids investigated here produce more compounds, significantly different ratios (% of odour compounds in the total blend), as well as new compounds in their floral odour compared to their progenitors. When tested for their attractiveness to the pollinator of each parent orchid species, we found that floral scent extracts of the hybrids triggered less inspecting flights and contacts by the male bees with the scented dummy than those of the parental orchid species. However, a series of additional behavioural bioassays revealed that the novel floral scent of the hybrids was significantly more attractive than either of the two parents to a pollinator species not initially involved in the pollination of any of the parent *Ophrys *species.

**Conclusions:**

Collectively, our results illustrate that the process of hybridisation can lead to the generation of evolutionary novelties, and that novel combinations of floral traits can drive pollinator shifts and rapid reproductive isolation in highly specific plant-pollinator interactions.

## Background

Angiosperms and their insect pollinators have flourished with extraordinary diversity through parallel and successive "explosive" radiations over the past 140 million years. It has been suggested that the intimate relationships between flowering plants and their pollinators have fuelled each other's diversification [1,2] and led to some of today's textbook cases of pollinator-mediated radiation such as in the Polemoniaceae family [3]. The examination of plant-pollinator interactions indicates that discrete floral differences among closely-related plant species can induce assortative pollinator attraction and contribute to reproductive isolation [4-7]. The origin of floral novelties (changes in the floral design, display, flowering time, chemistry and/or reward type) can therefore be considered as an important driving force in the diversification of flowering plants. Differences in floral phenotype can be generated by allelic variation at sometimes only one or a few loci [8-10], yet it has been shown that more "emergent" floral novelties (i.e., not predictable from the parents' phenotypes) can also originate *via *other processes such as polyploidy [11-14] or hybridisation between sympatric taxa [15]. Hybridisation can affect several phenotypic traits and niche dimensions which made this phenomenon and its creative potential a particularly important driving force in angiosperm evolution and diversification [15-23]. The acquisition of novel combinations of floral traits can help recombinant hybrids invade a vacant pollinator "niche", unexploited by its progenitors, which represents one route to adaptive evolutionary divergence, sympatric establishment and in some instances the origin of new species [18,24-30].

The orchid family and its ca. 24,000 species described to date (World Orchid Checklist, Royal Botanical Gardens Kew, UK) represent a particularly attractive group of flowering plants for studies addressing the ecological and evolutionary consequences of hybridisation. Indeed, the unusually high degree of specificity in pollinator attraction [31-35] and the apparent frequent formation of hybrids between orchid species, genera and even subtribes [36-38] offer a fertile loam for studies on the role of floral traits, including floral scents, in pollinator attraction. This is particularly true for some of the species-rich genera of so-called sexually deceptive orchids, like the European genus *Ophrys*, where pollinator attraction is brought about by a form of floral mimicry known as sexual deception. In this plant-pollinator interaction, a range of male insects (mainly wild bees, wasps and sometimes even beetles) pollinate the flowers during an attempted copulation (i.e., *pseudocopulation*) on the female decoys on the labellum [39,40]. Although floral colours/contrasts might be important in the detection of the flowers, the major floral attractant in this mimicry system is the floral scent, which mimics the female sex pheromone bouquet of a narrow range of targeted insect species [41-48]. Since sex pheromone communication channels are usually species-specific [[49], but see [50,51]], most *Ophrys *species are *de facto *reproductively isolated through the species-specific attraction of only one or a few closely related insect taxa [35,52,53]. Furthermore, when sympatric *Ophrys *taxa share the same pollinator species, cross-pollination is usually prevented by the attachment of the pollen masses (i.e., the pollinaria) of the orchid on different body parts of the insect (e.g. on the head vs. the abdomen tip) during pseudocopulation [39,40]. However, despite the apparent strength of pollinator-mediated reproductive isolation in *Ophrys *[54], a considerable proportion of these orchids that clearly belong to different and diagnosable species do hybridise in nature [55-59]. Hybridisation in *Ophrys *therefore provides unique opportunities for the formation of novel combinations of floral traits, particularly the composition of the floral scent, that can potentially drive shifts in pollinator niches and hence the rapid evolution of reproductive isolation between the hybrids and their sympatric parent species [see also [53]].

In this paper, we investigated the potential of *Ophrys *hybridisation to generate novel combinations of floral traits that could induce a pollinator shift. We used a combination of comparative chemical analyses of floral scents, genetic analyses of orchid taxa with AFLP molecular markers, *in situ *hand pollinations as well as behavioural bioassays with pollinators to uncover the origin and evolutionary consequences of hybridisation. Specifically, we ask the following questions: (i) How did the hybrids originate?; (ii) Are there differences in floral scent composition between the parental orchid species and the hybrids?; (iii) Can these floral differences induce a "niche shift" in the hybrids towards the attraction of a "new" pollinator species not exploited by any of the parental orchid species?

## Results

### Behavioural experiments with fresh inflorescences

**Figure 1:**
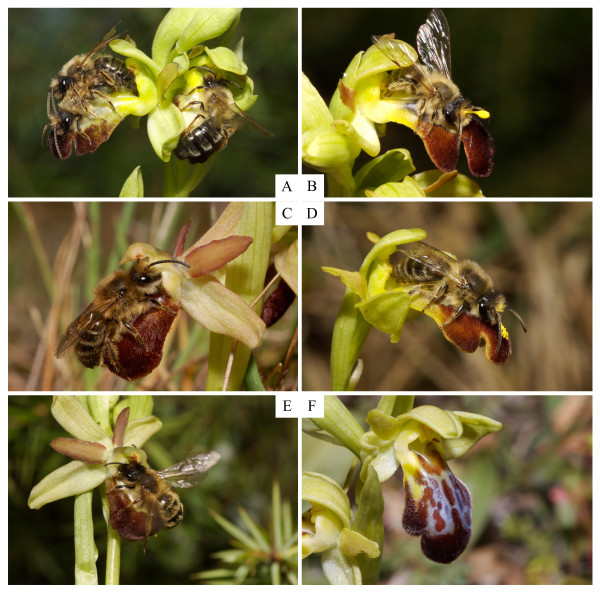
**Orchids and their pollinators in the hybrid zone**. A. Pseudocopulating males of *Andrena nigroaenea *(Kirby) (Hymenoptera, Andrenidae) in both the "abdominal" and the "cephalic" positions on the flower labella of *Ophrys lupercalis *Devillers-Terschuren & Devillers; B. Pseudocopulating male of *A. nigroaenea *in the "abdominal" position on a flower of *O. lupercalis *with pollinaria on its head; C. Pseudocopulating male of *Colletes cunicularius *(L.) (Hymenoptera, Colletidae) in the "cephalic" position on the flower labellum of *O. arachnitiformis*; D. Pseudocopulating male of *C. cunicularius *on the flower labellum of *O. lupercalis *with pollinaria on its head; E. Pseudocopulating male of *A. nigroaenea *in "cephalic" position on the flower labellum of *O. arachnitiformis*; F. A detail of a flower of the natural hybrid between *O. arachnitiformis *and *O. lupercalis *at the study site in southern France. All photographs by NJ Vereecken.

Our observations of pollinator behaviour during pseudocopulations with fresh, unpollinated flowers of the orchid taxa illustrate that the pollinators of each parent taxon systematically initiated copulation attempts on the orchid labellum in the expected position (i.e., "abdominal" on *O. lupercalis *and "cephalic" on *O. arachnitiformis*). However, we have observed that the insects' copulatory activity on the flowers regularly led to changes in their position on the labellum and occasionally to the subsequent uptake of pollinaria when in the alternative position (Figure 1). The supplementary video material [Additional file 1: Video] shows a male *C. cunicularius *pseudocopulating on the labellum of *O. lupercalis*, the other parent species, and withdrawing pollinaria on both its head and its abdomen tip during a single visit on the flowers.

### Behavioural experiments with floral odour extracts

**Figure 2:**
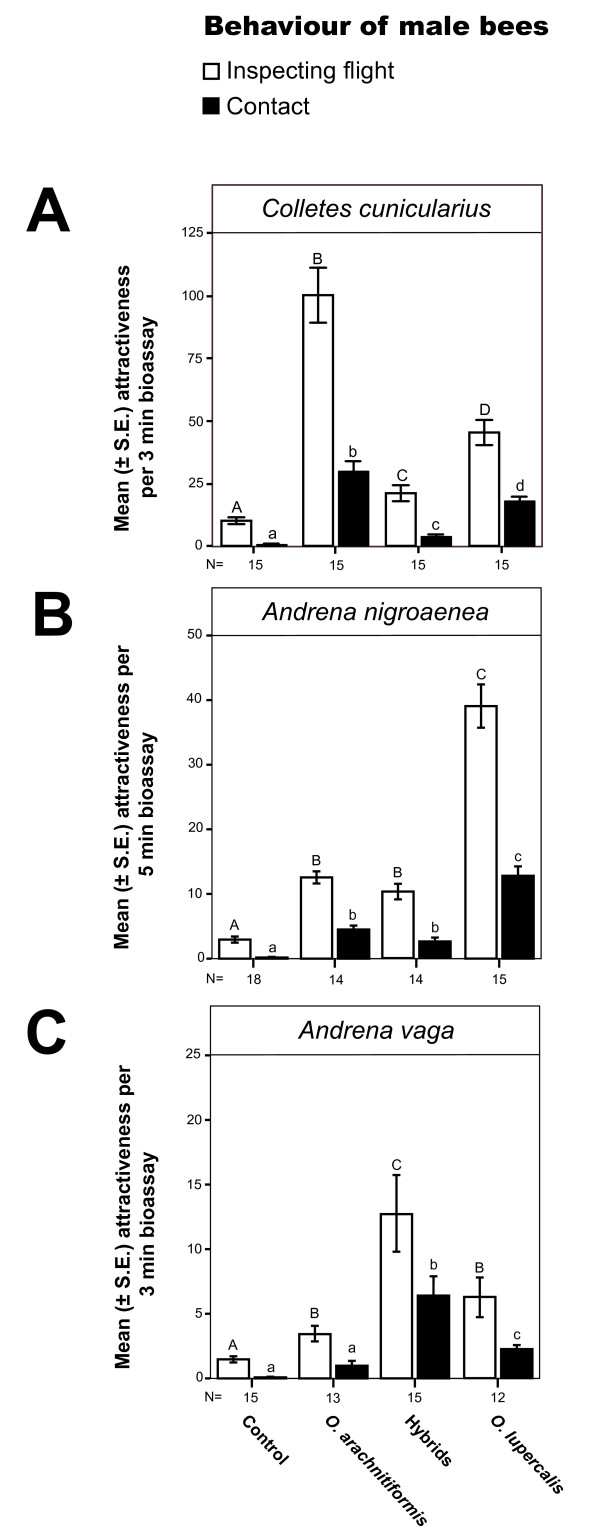
**Behavioural bioassays with the pollinators**. Comparative level of attractiveness of floral odour extracts of each taxa in the hybrid zone towards patrolling male bees. **A**. *Colletes cunicularius*, the pollinator of *Ophrys arachnitiformis*; **B**. *Andrena nigroaenea*, the pollinator of *Ophrys lupercalis*; **C**. *Andrena vaga*. One-Way ANOVA with LSD post-hoc test (a = 0.05). Different superscript letters on top of error bars indicate significant differences; the number of replicates is listed underneath the columns.

Results from our bioassays provide evidence for cross-attraction among orchid taxa towards patrolling males of *C. cunicularius *and *A. nigroaenea *(Figures 1 and 2). Specifically, we found that for each pollinator, floral odour extracts of the most commonly associated *Ophrys *taxa (e.g. *O. arachnitiformis *for males of *C. cunicularius*) were more attractive than floral odour extracts of the other parent species. The results from these bioassays further show that the floral odour extracts of hybrids triggered significantly less inspecting flights and contacts than those of the two parental species (Mann-Whitney *U*-test, *P *< 0.01), except for the bioassays with *A. nigroaenea *where no significant difference in attractiveness was found (Mann-Whitney *U*-test, *P *= 0.263) between the attractiveness of male bees towards floral odour extracts of the hybrids and of *O. arachnitiformis *(Figure 2). By performing bioassays with males of *A. vaga*, a species not initially involved in the pollination of the parent *Ophrys *species, we found that the floral odour of the hybrids triggered significantly more approaching flights to and contacts with the odour source than the floral odour of any of the two parents (Figure 2) (Mann-Whitney *U*-test, *P *< 0.01).

### Floral odour differentiation - hybrids and their progenitors

Our analyses of floral odour extracts of each orchid taxa in the hybrid zone have detected the presence of 80 individual compounds in total, including the biologically active compounds identified for *A. nigroaenea*, the pollinator of *O. lupercalis *[60,61], and for *C. cunicularius*, the pollinator of *O. arachnitiformis *(= *O. exaltata*) [44- Additional file 2: Supplemental Table S1]. A comparison of the number of floral odour compounds found in each taxon is provided in Table 1. With a total of 73 odour compounds, the hybrids produce more odour compounds (qualitatively) in their floral odour than any of their parent species. We also found some compounds to be taxon-specific, in particular 2 "new" odour compounds produced exclusively by the hybrids whose GC-MS spectra indicate that they are straight-chained alkenes (mono-unsaturated alkenes, position of the double bound unknown) of 23 and 25 carbon chain length, respectively.

**Table 1:** Floral odour chemistry in the hybrid zone

Orchid taxa	Number of floral odour compounds identified (% of total)	Number of taxon-specific odour compounds identified
*Ophrys arachnitiformis*	64 (80%)	3

Hybrids	73 (91.25%)	2

*Ophrys lupercalis*	66 (82.5%)	1

Summary of floral odour compound production in each orchid taxon of the hybrid zone investigated. A total of 80 odour compounds have been identified, 48 (i.e. 60%) of which were produced by all three taxa simultaneously.

**Figure 3:**
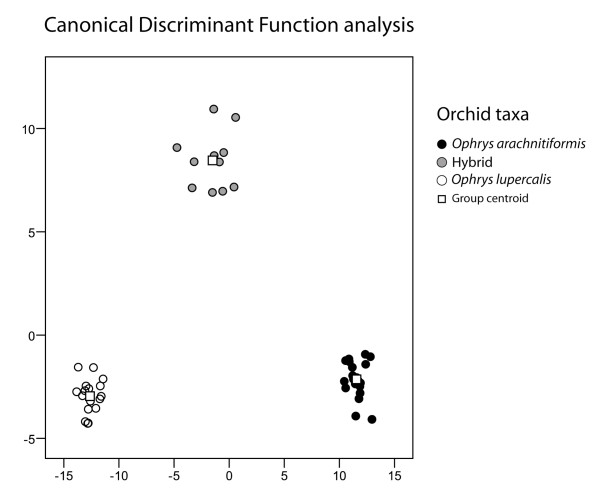
**Floral scent profiles**. Floral scent differentiation among taxa in the *Ophrys *hybrid zone investigated. Canonical discriminant function (CDF) plot of all odour compounds (relative proportions, in %) found in epicuticular extracts of the *Ophyrs *flowers. Functions 1 and 2 account for 100% (86.0% and 14.0%, respectively) of the total variability in floral odour among orchid taxa.

A canonical discriminant function (CDF) analysis of odour compounds resolved the hybrids and their progenitor species into non-overlapping groups (Figure 3). This CDF analysis rejects the null hypothesis of homogeneity of covariance matrices (small Wilks' λ values: Wλ_1 _= 0.000002; Wλ_2 _= 0.042 and associated P_1 _and P_2 _< 0.001). The high discriminatory ability of the canonical discriminant functions 1 and 2 plotted in Figure 3 provide evidence for the importance of the independent variables (i.e., all floral odour compounds) to the discriminant analysis. Canonical correlation values close to 1 (Cc_1 _= 0.996; Cc_2 _= 0.979) associated with the two CDF's plotted further account for the significant contribution of the first two canonical discriminant functions to the resolving of all three orchid taxa into non-overlapping groups. The CDF's 1 and 2 account for 100% (84.0% and 16.0%, respectively) of the total odour variance among orchid taxa, which further indicates their great discriminatory ability in the model (100% of cross-validated grouped cases were correctly classified). Overall, 97.9% of all cross-validated samples were assigned correctly to their taxa by the two CDF's (*O. arachnitiformis*: 100%; Hybrids: 90.9%; *O. lupercalis*: 100%). The analysis of the partitioning of the floral odour variance yielded a significantly higher proportion of the variance among taxa (65%) over within taxa (35%).

**Figure 4:**
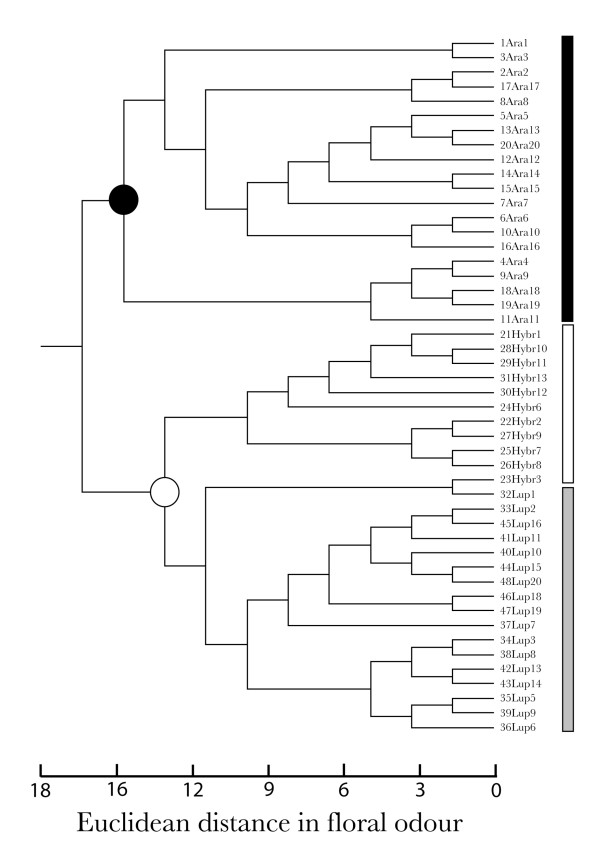
**Floral scent clustering**. Floral odour similarity between samples of *Ophrys arachnitiformis *(solid, black bar), hybrids (open bar) and *O. lupercalis *(solid, grey bar) in the orchid hybrid zone. The UPGMA cladogram was based on pairwise Euclidean distance in floral odour (relative proportions of all compounds, in %) between samples. Individuals are grouped together according to taxa (except for one sample, labelled "23Hybr3"), and hybrids cluster together with *O. lupercalis *(open circle at internal node), whereas samples of *O. arachnitiformis *cluster separately (solid circle at internal node).

The analysis of overall floral odour similarity (relative proportions of all compounds, in %) among samples was performed using unweighted pair groups with arithmetic averages (UPGMA). Our results show that, besides the fact that all but one sample ("23Hybr3" on Figure 4) grouped together according to taxa, the floral odour of the hybrids is chemically asymmetric towards *O. lupercalis*. The UPGMA cladogram revealed two discrete clusters, the first one comprising all the samples of *O. arachnitiformis *(solid circle at internal node, Figure 4) and the second consisting of two subclusters containing *O. lupercalis *and the hybrids (open circle at internal node, Figure 4).

### Molecular hybrid index scores

**Figure 5:**
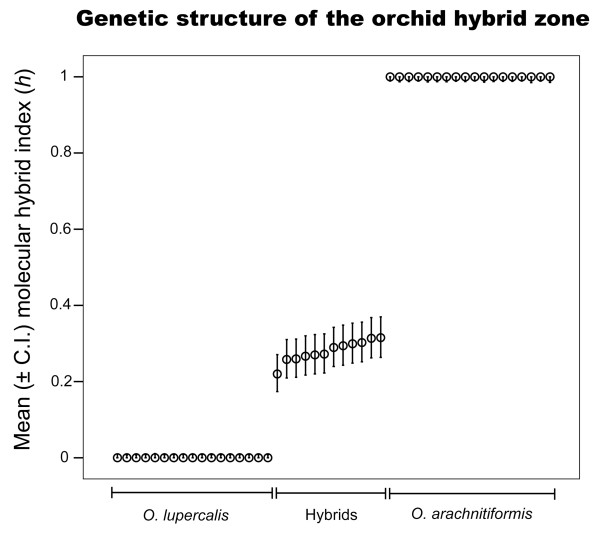
**Genetic architecture of the hybrid zone**. Maximum-likelihood estimates of molecular hybrid indices (MLE(*h*)) and their associated confidence intervals based on band frequencies for all individuals (both parental species and their hybrids) sampled across the hybrid zone. The hybrid index (*h*) ranges from zero to one, corresponding to pure individuals of *Ophrys lupercalis *(the other parent species) and *O. arachnitiformis *(the reference species), respectively.

The eight primer pair combinations used for AFLP analysis produced a total of 390 polymorphic markers. Using the 100% difference criterion, we identified 157 markers as species-specific for *Ophrys arachnitiformis *and *O. lupercalis*. Of these, 95 were present as bands in *O. arachnitiformis *and 62 in *O. lupercalis*. The hybrid index analysis to allocated individuals to the specific hybrid class with high confidence (Figure 5). The hybrid index analysis based on species-specific markers revealed exactly the same pattern of hybrid index analysis calculated with all polymorphic markers (data not shown). The mean ML hybrid index (MLI(*h*)) for hybrid individuals was 0.277 (S.E. ± 0.008) with diagnostic species-specific markers. We found no evidence for patterns of genetic variation suggestive of gene introgression into parental classes in the hybrid zone investigated (Figure 5).

### Crossing experiments and flow cytometry analyses

None of the hand-pollinations performed between the hybrids and the parents resulted in capsule formation. Hand-pollinations between hybrids yielded only one capsule that contained few seeds that were devoid of embryo, which we therefore considered unviable. We did not check whether the pollen tubes made it all the way to the ovules in the back-crosses and the F1xF1 crosses.

The flow cytometry profiles produced consistent results within taxa and revealed differences in ploidy level among the *Ophrys *species investigated. Our analyses of pollinia and leaf fragments showed that *O. bilunulata *(control 1), *O. sphegodes *(control 2) and *O. arachnitiformis *(parent 1) have the same ploidy level, namely that they are all diploid (2n = 36). The samples of *O. lupercalis*, however, contained twice as much DNA as those of the aforementioned species, which suggests that all the *O. lupercalis *individuals investigated in the hybrid zone are tetraploid (4n = 72). Finally, the analyses of DNA contents of both pollinia and leaf fragments of the hybrids revealed a ploidy level intermediate to that of the two parents, which supports the scenario that the hybrids sampled in the hybrid zone are triploid (3n = 54).

## Discussion

### The origin of *Ophrys *hybrids

Our study provided evidence for the occasional breakdown of both ethological and mechanical isolation barriers mediated by the pollinators, which provides opportunities for the formation of hybrids between *O. arachnitiformis *and *O. lupercalis *when they are found in sympatry. The cross-attraction of each parent orchid species to each pollinator was allowed by the emission of (at least partly) overlapping patterns of biologically-active compounds for each pollinator species [Additional file 2: Supplemental Table S1]. It is generally assumed that sexually deceptive orchids are strongly isolated *via *pre-pollination barriers, and more weakly through post-zygotic (i.e., post-pollination) mechanisms, which contrasts to the situation observed in food deceptive orchids that have weak pre-pollination [62] but strong post-zygotic isolation barriers [63]. Yet in this study, we have been able to show that pre-pollination (ethological and mechanical) isolation barriers, which are generally thought to represent strong isolation mechanisms in flowering plants, especially when they act in concert (e.g. [64]), could be more permeable than previously thought in the genus *Ophrys *(Figure 1 and [Additional file 1: Video]).

Many species of solitary bees that act as pollinators in *Ophrys *use specific female sex pheromone that are often based on a "variation on a theme", i.e. identical compounds in different ratios [53,57,65]. This phenomenon enhances the probability for an *Ophrys *species to be cross-attractive to different pollinators, even though the flowers might receive significantly more visits by their most commonly associated pollinator (see Figure 2). Hence, reproductive isolation is usually maintained but does not prevent opportunities for gene flow between sympatric species, at least theoretically. Besides, the attractiveness of orchids to alternative pollinators observed in *O. arachnitiformis *and *O. lupercalis *(Figures 1 and 2) might enhance the reproductive output of these orchids at a local scale, a mechanism that should be favoured by selection since orchids in general and deceptive ones in particular are often pollinator-limited in their reproductive success [34,42,66,67].

### Hybrid floral novelty and pollinator shift

A prerequisite for adaptive evolutionary divergence of hybrids is the invasion of an alternative "niche" in which the hybrids are subjected to different selection pressures, and the parallel evolution of reproductive isolation between the parents and the hybrids. The results from our analyses and bioassays support these two requirements, first because we have shown that the F1 hybrids emit "emergent" combinations of floral scent compouds, i.e. they are not intermediate between their parents or have the sum of their parents' traits, but instead they have developed an "emergent" floral scent novelty with completely new compounds that are not predictable from the parents' phenotypes. Second, this floral scent novelty leads to the acquisition of a novel pollinator "niche" by hybrids (Figure 3, Table 1) that can drive the rapid evolution of reproductive isolation between the hybrids and their parents (Figure 2). These data are consistent with previous studies reporting that even small changes in floral scent chemistry in *Ophrys *have the potential to mediate assortative pollinator attraction [53,65] and hence promote rapid reproductive isolation between diverging sympatric orchid taxa if maintained over generations [68]. Furthermore, our results challenge the view that hybrids often experience reduced fitness compared to their parents [57,69-72] by showing that they can also outcompete their parents under certain ecological conditions (here, different pollinator environments, but see also [73]). Finally, the last major step towards fully autonomous establishment requires the hybrids to be fertile [22,25]. Here, none of our bidirectional and controlled hand-pollinations resulted in capsule formation, and hand-pollinations within the group of hybrids yielded only one capsule that contained seeds devoid of embryo, which suggests that the hybrids tested here between *O. arachnitiformis *and *O. lupercalis *are both maternally and paternally sterile, a likely consequence of their triploidy.

Although the triploid hybrids investigated have been shown to be sterile, their evolutionary potential should not be dismissed *a priori*. Indeed, studies on allotriploids obtained *via *diploid and tetraploid parent species indicate that many of the gametes produced by triploids are not functional, because they possess aneuploid, unbalanced chromosome numbers. However, triploids may occasionally generate small numbers of euploid (x, 2x) gametes and they can also produce 3x gametes via non-reduction (reviewed by [74]). This may enable allotriploids to produce fertile allotetraploids by selfing or backcrossing with diploid parents [75-78] without otherwise significant changes in the genomic architecture of the polyploid individuals [79]. Several studies have shown that massive expression changes often accompany polyploid formation but autopolyploidy might produce less dramatic expression changes than allopolyploidy. hybridisation is likely to cause more dramatic phenotypic changes than genome doubling *per se *because it results in transcriptional effects following the combination of differentiated genomes, with their divergent regulatory machinery, into a common nucleus (reviewed in [80]). If filled, these requirements could potentially open up a new route to the emergence of a hybrid neospecies through the attraction of a novel pollinator, provided potential pollinators are present locally. This scenario could also help explain the occurrence of several endemic species of *Ophrys *frequently characterised by a tetraploid genome [81].

The parental species pair investigated in this study turned out to have different ploidy levels, yet reports indicate that *O. lupercalis *might be diploid in other regions of the Mediterranean Basin [82]. These contact sites between *O. arachnitiformis *and *O. lupercalis *should be investigated by using the present study as a conceptual touchstone to ultimately determine whether or not the local hybrids are at least partially fertile. Other sympatric species of *Ophrys *that have the same ploidy level are known to produce hybrids that set seed when they are experimentally pollinated with pollinia from other sympatric hybrids [57,58]. Among these hybrids, several have been reported to have a significantly different floral odour bouquet compared to their parents [83]. Our results should therefore encourage investigations in these hybrid zones to test the extent to which hybrids can escape the ecological niche of their parents through the attraction of pollinators that are not involved in the pollination of their parents [19,21,25,84].

## Conclusions

In this study, we have found evidence that hybridisation in sexually deceptive orchids can produce novel combinations of floral traits, particularly their floral odour (Figures 3 and 4), that can in turn lead to a pollinator shift (Figure 2). However, the development of the hybrids under study into novel "ecological" species is hindered by their sterility, which is also expected to impede the formation of later-generation progeny via selfing, sib-crossing with neighbouring hybrid individuals, or back-crossing with any of the sympatric parental species. Yet, although several assumptions are not met for making plausible ecological speciation by hybridisation in the hybrids between *O. arachnitiformis *and *O. lupercalis*, our study provides a unique window into the stepwise process by which apparent reproductive barriers can be broken down and how new combinations of floral traits can be generated, leading to the evolution of novel, highly specific plant-pollinator interactions.

## Methods

### Species profiles

The model organisms chosen in this study are *Ophrys arachnitiformis *and *O. lupercalis*, two species that belong to different sections within the genus *Ophrys *(sections *Euophrys *and *Pseudophrys*, respectively). These taxa often bloom and grow in sympatry from mid-March to mid-April in southern France where they are pollinated by males of *Colletes cunicularius *and *Andrena nigroaenea*, respectively. During pseudocopulation, the orchids' pollinaria are attached on distinct body parts of the pollinator (on the face for *C. cunicularius *and on the abdominal tip for *A. nigroaenea*). Hence reproductive isolation between *O. arachnitiformis *and *O. lupercalis *growing in sympatry is usually achieved through a combination of (i) ethological isolation (the specific attraction of distinct pollinator taxa) and (ii) mechanical isolation (the differential attachment of pollen masses on the body of their respective pollinator). However, these orchid species were found to hybridise at Torreilles (S-France) in a population where only the two aforementioned *Ophrys *species and their hybrids grew intermixed. These hybrids between these parent species are rare in the wild and when formed, they are mostly found at low densities in populations largely dominated by the parent species.

The geographic range of the solitary bee *A. vaga *extends from middle and north Europe to Central Asia [85], and in France down to the Rhône river valley [86] where both *O. arachnitiformis *and *O. lupercalis *are known to occur [87].

### Sample collection and preparation

A total of 53 individuals representative of each orchid taxa in the hybrid zone (*O. arachnitiformis *(20); hybrids (13); *O. lupercalis *(20)) were labelled individually and sampled for chemical analyses of their floral odour; the same individuals were used to sample plant material for genetic analyses. Individual labella of fresh, unpollinated *Ophrys *flowers of both parent species and their hybrids were extracted in 200 μl of hexane (HPLC grade) for one minute. All floral extracts were stored at -20°C for subsequent gas chromatography (GC) analyses and behavioural bioassays.

### Chemical analyses

All samples were analysed by gas chromatography (GC) on a Hewlett Packard 6890N GC equipped with a HP-5 capillary column (30 m * 0.32 mm * 0.25 μm). The injector temperature was kept at 300°C. One μL aliquots of the extracts were injected splitless at 50°C (1 minute), followed by a programmed increase of oven temperature to 300°C at a rate of 10°C/min; helium was used as the carrier gas. 100 ng of *n*-octadecane was added as an internal standard to each sample. Compounds were identified by comparison of retention times with authentic standard compounds, and a selection of samples were analysed with a Finnigan Trace Ultra GC coupled with a Finnigan POLARIS Q ion trap mass spectrometer under the temperature conditions mentioned above. The absolute amounts of all 80 identified compounds were calculated by the internal standard method as described by Mant et al. [44]. Relative proportions (%) were calculated by summing up the absolute amounts of all compounds; absolute amounts of individual compounds were then divided by the total and multiplied by 100.

### Behavioural experiments - fresh inflorescences

During this study, we have performed behavioural observations of the pollinators using fresh, unpollinated flowers of *Ophrys arachnitiformis *and *O. lupercalis*. Pollinators were observed *in situ*, caught and identified. Pollinator behaviour on the flowers was recorded using macro photography of pseudocopulating bees in order to determine if both pollinators are able to transfer pollinaria between the parent orchid species during pseudocopulations.

### Behavioural experiments - floral odour extracts

To quantify the relative attractiveness of orchid taxa in the hybrid zone, we have performed behavioural bioassays with patrolling male bees using dummies scented with solvent extracts of unpollinated flowers of each orchid taxa in the hybrid zone. The orchid species investigated are flowering in very early spring, at a time when only very few solitary bees have emerged from their underground cell. Picked inflorescences of the hybrids were tested for their attractiveness towards 5 species of solitary bees (*Andrena bicolor, A. flavipes, A. vaga, Anthophora plumipes *and *Eucera elongatula*) that could potentially act as pollinators since they were active at the same time of the year (in early March) and their distribution range overlaps with that of the orchids investigated. Among these solitary bee species, only the males of *A. vaga *seemed to be interested in the hybrids and they were observed visiting the flowers frenetically. These preliminary observations have led to the choice of this species as a potential pollinator of the hybrids and the subsequent series of bioassays performed in the field. All behavioural bioassays were performed in late March and early April 2006-2007 in natural populations of *Colletes cunicularius *and *Andrena nigroaenea *in Cadillon (southern France) as well as in an allopatric natural population of *A. vaga *(Braine-l'Alleud, Belgium) where male bees were patrolling for emerging females on restricted nesting/emergence sites. The density of bees in each site was stable over the days of observations. These bee species rank among the first bees to emerge in early spring and their flight period largely overlaps with that of the three orchid taxa investigated. Behavioural responses of male bees towards scented dummies (black cylindrical plastic beads, 4 × 5 mm, mounted on an insect pin) were taped using a digital voice recorder (during 3 minutes for *C. cunicularius *and *A. vaga*, 5 minutes for *A. nigroaenea *due to a lower population density and activity) and classified in two categories: (i) number of approaches (inspecting flight in front of the dummy at close range (< 10 cm) without any contact with the odour source), and (ii) number of contacts with the scented dummy. Odour samples were tested individually and each scented dummy was used only once. Half the amount of each natural extract was applied on each dummy with a Hamilton glass syringe (100 μL) for each behavioural bioassay, whereas the second half was saved for chemical analyses. The dummy was placed in a male patrolling area after the solvent had evaporated. Controls (dummies treated with solvent only) were tested successively for their attractiveness after every 5th test. All bioassays were conducted between 10 a.m. and 3 p.m. - when patrolling activity of male bees was at its peak. Since males of *C. cunicularius *are known to patrol fairly localised regions on the nesting/emergence site [87] and since the same phenomenon is likely to be observed in the other bee species investigated, test spots were changed after each bioassay to test the responses of different males to natural extracts of *Ophrys *flowers and to avoid any habituation of the male bees to the test spots.

### DNA extraction and molecular analyses

For each orchid taxa, a leaf fragment of ca. 8 cm^2 ^was excised and the plant tissue was desiccated using silicagel, each in individual sealed plastic bags. Genomic DNA was extracted using a slight modification of CTAB protocol of Doyle & Doyle [88]. Plant leaf material was macerated in 700 μL of standard CTAB buffer, incubated at 60°C for 30 min, extracted twice with chloroform-isoamyl alcohol, precipitated with isopropanol and washed with 70% ethanol. Precipitated DNA was then resuspended in 50 μL of distilled water. Amplified Fragment Length Polymorphism (AFLP) analysis was performed using a modified version of Vos et al. [89]. Restriction-digestion was conducted using restriction enzymes EcoRI and MseI on 300 ng genomic DNA. Ligation of EcoRI and MseI adapters to restriction fragments took place concurrently with restriction digestion. A pre-amplification PCR of the restriction fragments was conducted using a template of 2 μl of the restriction-ligation product. Primers for the preamplification were EcoRI and MseI primers with one additional selective nucleotide. A second selective amplification was conducted with 1 μl of preamplification product, primers were the same as in preamplification, but with two or three additional selective nucleotides. A total of six primer pairs were used. Fragment separation and detection took place on ABI 3130 AVANT DNA sequencer. Fragment sizes (in bp) were determined with the software Genemapper 3.7 by using an internal size standard (GeneScan Rox500, Applied Biosystem).

### Molecular data analysis

The AFLP profiles generated using the various primer combinations were scored in terms of presence or absence of each marker in each individual plant. Fragments were always scored as dominant markers excluding monomorphic markers from all further analysis. The genetic marker data were used to calculate a molecular hybrid index for each individual. To estimate the hybrid index we used the software HINDEX, which applies a maximum-likelihood estimate approach [90]. The hybrid index (*h*) ranges between zero and one, corresponding to pure individuals of the alternative and reference species, respectively [90]. We calculated the hybrid index both using all polymorphic markers and the species-specific marker only. We considered markers as species-specific if they occurred in 100% of the individuals in one parental species, but were absent from the other parental species.

### Crossing experiments and flow cytometry analyses

Five hybrid individuals, representing a set of 15 fresh and unpollinated flowers, were dug out, transplanted in pots and used for hand pollination experiments. Each flower was cross-pollinated using one pollen mass from another hybrid individual. Additionally, 20 hybrid pollinaria were used for hand pollinations with each parent (10 hand pollinations for each hybrid-parent pair). All the hand-pollinated individuals transferred in pots were stored in a temperate greenhouse until the inflorescences wilted and the formation of fruits (capsules) was complete. We then counted the number of capsules formed and examined the seeds contained in the capsules for the presence of embryos by light microscopy.

To assess the relative ploidy level of *Ophrys arachnitiformis*, *O. lupercalis *and their hybrids in the hybrid zone investigated, we have performed flow cytometry analyses using a PA-I flow cytometer with HBO (high pressure mercury lamp) PARTEC^© ^(Partec Gmbh, Münster, Germany). Plant material (fresh leaf fragments and pollinia) of *O. bilunulata*, a diploid, closely-related species of *O. lupercalis *[82,87], and of *O. sphegodes *were used as controls for the calibration of the flow cytometer.

### Statistic analyses

We used multivariate analyses to investigate floral odour differentiation (relative amounts of all compounds, in %) among the orchid taxa in the hybrid zone. First, we used a principal component analysis (PCA) to reduce the number of variables (odour compounds) in the analysis. All 15 principal components generated by the PCA were then used in a canonical discriminant function (CDF) analysis. We used all the floral odour compounds recorded in solvent extracts since the data did not contain significant outliers and given that this multivariate method is robust even when the homogeneity of variances assumption is not met [91]. We used a pairwise individual-by-individual Euclidean distance matrix (calculated from the relative amounts of odour compounds in SPSS 13.0) as input file in GenAlEx 6 [92] to analyse the partitioning of odour variance among and within orchid taxa. This analysis is based on an adaptation of the AMOVA framework for the analysis of odour (see [68] for additional details); random permutations (n = 99) were used to test for significant differences in odour partitioning among species. The individual-by-individual Euclidean distance matrix was also transferred to PAUP* 4.0 [93] to construct an unrooted UPGMA tree to depict the floral odour similarities among individuals and taxa in the hybrid zone. To test for differences in male bee responses to natural extracts of *Ophrys *flowers during the behavioural bioassays, a Kruskall-Wallis test and pairwise Mann-Whitney *U*-tests were used with the level of significance (α) set at 0.05. All these statistical tests were performed with the SPSS 13.0 software [91].

## Authors' contributions

NJV carried out the behavioural experiments, chemical, statistic, and flow cytometry analyses, manual crosses and drafted the manuscript. SC and his team carried out the molecular analyses and helped to draft the manuscript. FPS contributed to chemical reagents and analyses, and helped to draft the manuscript. All authors read and approved the final manuscript.

## Supplementary Material

Additional file 1 **Video**. The video depicts a male of the solitary bee *Colletes cunicularius *pseudocopulating on the flower labellum of *Ophrys lupercalis*: pollinia removal on the abdomen tip and on the head of the pollinator (video courtesy of J-C Milhé). Click here for file

Additional file 2 **Table S1 - Floral odour chemistry in the hybrid zone**. Summary of the qualitative differences in floral odour compound production in each orchid taxon of the hybrid zone investigated. Click here for file
