# Supplementary material for: Hybrid floral scent novelty drives pollinator shift in sexually deceptive orchids
Source: BMC Evol Biol. 2010 Apr 21;10:103. doi: 10.1186/1471-2148-10-103 (PMC2875231; doi:10.1186/1471-2148-10-103)
Supplement: Additional file 2 — Table S1 - Floral odour chemistry in the hybrid zone. Summary of the qualitative differences in floral odour compound production in each orchid taxon of the hybrid zone investigated. [file 1471-2148-10-103-S2.PDF]

STable 1. Comparison of patterns of biologically-active (A) vs. non-active compounds (NA) for *Colletes cunicularius* and *Andrena nigroaenea* males identified in the floral scent of *Ophrys arachniformis* and *O. lupercalis*. a. Results after [44]; b. Results after [60, 65]; *not referred to in results.

		Colletes cunicularius [a]	*Andrena nigroaenea* [b]
	**Aliphatic hydrocarbons - alkanes/alkenes/alkadienes**		
1.	Heneicosane	A	A
2.	Docosane	NA	A
3.	Tricosane	A	A
4.	Tetracosane	NA	A
5.	Pentacosane	A	A
6.	Hexacosane	NA	A
7.	Heptacosane	A	A
8.	Octacosane	NA	NA*
9.	Nonacosane	NA	NA*
10.	(*Z*)-7-Heneicosene	A	NA*
11.	(*Z*)-7-Tricosene	A	NA*
12.	(*Z*)-5-Tricosene	NA	NA*
13.	(*Z*)-3-Tricosene	NA	NA*
14.	(*Z*)-9-Tetracosene	A	NA*
15.	(*Z*)-12/(*Z*)-11-Pentacosene	NA*	A
16.	(*Z*)-9-Pentacosene	A	NA*
17.	(*Z*)-7-Pentacosene	A	NA*
18.	(*Z*)-5-Pentacosene	NA	NA*
19.	Heptacosadiene	NA*	A
20.	(*Z*)-13/(*Z*)-12/(*Z*)-11-Heptacosene	NA*	A
21.	(*Z*)-9-Heptacosene	A	A
22.	(*Z*)-7-Heptacosene	NA	A
23.	Nonacosadiene	NA*	A
24.	(*Z*)-8, (*Z*)-20-Nonacosadiene	A	NA*
25.	(*Z*)-14/(*Z*)-13/(*Z*)-12/(*Z*)-11-Nonacosene	NA	A
26.	(*Z*)-11-Nonacosene	A	NA*
27.	(*Z*)-9-Nonacosene	A	A
28.	(*Z*)-7-Nonacosene	NA	NA*
29.	(*Z*)-8, (*Z*)-20-Hentriacontadiene	A	NA*
30.	Hentriacontadiene	NA*	A
31.	(*Z*)-9-Hentriacontene	A	NA*
	**Other compounds**		
32.	Linalool	A	NA*
33.	Nonanal	NA*	A
34.	Decanal	NA*	A
35.	Nonanoic acid	NA*	A
36.	Undecanal	NA*	A
37.	Dodecanal	NA*	A
38.	Tridecanal	NA*	A
39.	Hexadecanal	A	A
40.	Octadecanal	NA*	A
41.	Nonadecanal	NA*	A
42.	Eicosanal	A	A
43.	Heneicosanal	NA*	A
44.	Tetracosanal	NA	A
45.	Hexacosanal	NA*	A
46.	Oleic acid	NA	NA*
47.	Linoleic acid	NA	NA*
